# Future of Valvular Heart Disease and Structural Heart Interventions: Why So Much Excitement?

**DOI:** 10.3390/jpm15090443

**Published:** 2025-09-19

**Authors:** Eirini Beneki, Julia Grapsa

**Affiliations:** 1 Department of Cardiology, Lausanne University Hospital (CHUV), 1011 Lausanne, Switzerland; e.beneki@hotmail.com; 2 Department of Cardiology, Brigham and Women’s Hospital, Harvard Medical School, Boston, MA 02115, USA

**Keywords:** structural heart disease, valvular disease, imaging, interventions, artificial intelligence, multidisciplinary teamwork

## Abstract

Valvular heart disease (VHD) is becoming increasingly prevalent in the aging population and continues to be a major contributor to cardiovascular morbidity and mortality. Advances in non-invasive imaging, able to confirm the presence and severity of valve disease, have been crucial in revealing VHD mechanisms through the assessment of morphological and functional changes. In parallel, immense progress in both surgical techniques and catheter-based interventions has broadened therapeutic options, particularly for high-risk and elderly patients. Despite the availability of evidence-based guidelines, a shared decision-making process should play a key role in the final decision for therapy, outlining the goals and risks of possible intervention coupled with the patient’s own needs and expectations. Future research should aim to develop safer, more effective, and longer-lasting treatments tailored to the unique need of each patient with VHD.

## 1. Introduction

The field of valvular heart disease (VHD) has exploded over the last two decades, and there is much excitement about this progress from every frontier. The future is even brighter, with clinical practice transforming further as more research focuses on the complex molecular mechanisms that accelerate early diagnosis and precise management of these patients. Moreover, a major objective of current and future research should focus on increasing the longevity and long-term performance of bioprosthetic valves, ensuring they last longer and function reliably for patients over time.

Rapid advances in imaging, transcatheter technologies, computational modeling, and device engineering expand treatment options, especially for patients once deemed inoperable or high-risk. Increasingly, multidisciplinary heart teams are integrating novel techniques to personalize care, improve outcomes, and enhance quality of life. Together, these advances are fostering increased optimism about the future of VHD and structural heart disease (SHD) interventions, pushing the boundaries of what is possible, driving innovation, and setting new standards for how complex valve and structural heart conditions are managed worldwide.

## 2. Multimodality Imaging in Structural Heart Disease

Multimodality cardiac imaging advances have shed light on the pathophysiology of common VHDs and reclassified the severity of valvular diseases, leading to important prognostic implications. More specifically, imaging contributes to VHD diagnosis and treatment by identifying the valve lesion and quantifying the severity. Further assessment of valve dysfunction repercussion on cardiac function offers valuable information on prognosis. It contributes to the determination of optimal timing (watchful waiting or prophylactic) as well as the type (surgical or transcatheter valve replacement or repair) of valve procedure and helps in defining the follow-up period [1].

Echocardiography remains the primary imaging method for the diagnosis of VHD. Transthoracic echocardiography is a safe, non-invasive, and often portable method, enabling a detailed assessment of the underlying mechanism and severity of each valve lesion, as well as evaluation of ventricular performance, especially with the use of real-time three-dimensional imaging [2]. Other valuable information provided by echocardiography is left ventricular ejection fraction, which remains an important criterion for the decision of whether patients should be referred for surgery. 

Transesophageal echocardiography, although not routinely performed, is the next step in cases of diagnostic uncertainty of the severity of a valvular lesion with transthoracic echocardiography. Three-dimensional (3D) echocardiography helps in visualizing valve morphology, leaflet motion, and regurgitant jets. More specifically, 3D reconstruction with the application of digital heart models (DHMs) allows simulation of valve mechanics under physiological conditions. In the assessment of aortic stenosis, 3D imaging more accurately reproduces the anatomical geometry of the left ventricular outflow tract (LVOT) and aortic valve orifice compared with two-dimensional (2D) imaging, which relies on geometric assumptions. This facilitates direct planimetry of the aortic valve area (AVA). While DHM provides valuable anatomical detail and can improve measurement accuracy in borderline or discordant cases, it does not replace functional assessment using the continuity equation, which incorporates flow measurements from the LVOT and transvalvular velocities to calculate the effective AVA. Moreover, direct planimetry assessing the area of stenotic valves, especially in multivalvular disease cases, and multiplanar reconstruction with the use of real-time 3D transesophageal echocardiography can be valuable to measure mitral valvular annulus (MVA) in rheumatic mitral stenosis when Doppler measurements are not reliable due to concomitant aortic stenosis and regurgitation [3]. Hence, 3D echocardiography helps quantify valve opening area, especially in stenotic lesions, and assess ventricular–atrial interaction and functional consequences of valve disease. The application of real-time 3D echocardiography with color-defined planimetry in degenerative calcified mitral stenosis has also proven to be beneficial [4]. Additionally, the superior accuracy of 3D echocardiography in assessing ventricular volumes and ejection fraction compared with traditional 2D techniques makes it a valuable tool in the evaluation of valvular heart disease. Finally, offering real-time imaging of cardiac structures and devices, transesophageal echocardiography is also useful for the guidance of interventional procedures, such as transeptal puncture and valvular interventions, while potentially reducing radiation exposure from fluoroscopy [2].

Intracardiac echocardiography has been used to guide a limited number of transcatheter cardiac interventions. Recent advances in structural heart interventions have increased interest in intracardiac echocardiography as an alternative to transesophageal echocardiography, minimizing the need for general anesthesia and intubation [5]. In addition, intracardiac echocardiography offers several potential advantages, including superior visualization of specific structures (e.g., the pulmonary valve and the inferior portion of the interatrial septum), more efficient use of resources (e.g., personnel and catheterization laboratory time), and reductions in radiation exposure and hospital length of stay. Nevertheless, despite over a decade of experience, the limited availability of data and the relatively small number of operators proficient in this technique have constrained its broader adoption.

Stress echocardiography is recommended when resting hemodynamic and echocardiographic findings do not fully explain patient’s symptoms [6] and in non-severe lesions where exercise may exacerbate the hemodynamic impact of the predominant lesion and thereby provoke symptoms that are otherwise absent at rest. Dobutamine stress echocardiography is predominantly applied in low-flow, low-gradient aortic stenosis in patients with reduced ejection fraction in order to rule out pseudo-severe aortic stenosis.

Speckle-tracking echocardiography is widely recognized as one of the most valuable tools for diagnosing and predicting outcomes in VHD, thanks to its ability to detect subclinical myocardial dysfunction before a measurable decline in left ventricular ejection fraction occurs [7]. However, strain imaging can now detect impaired left ventricular systolic function before left ventricular ejection fraction reduces, thus provoking the debate on whether patients with severe VHD should be referred for surgery at an earlier stage, before the onset of symptoms. Impaired left ventricular strain has been proven to correlate with the amount of myocardial fibrosis detected with cardiac magnetic resonance (CMR) techniques, and the extent of fibrosis associated with severe VHD has important prognostic implications [8]. Studies on single VHD have already shown the prognostic impact of strain analysis in such patients, but scarce data do exist on the diagnostic and prognostic utility of speckle-tracking in multivalvular disease [9].

Although the Doppler echocardiography remains the primary imaging technique for the clinical management of VHD, other imaging modalities, such as multidetector computed tomography (CT) or CMR, are often useful to confirm the information obtained by Doppler echocardiography. Thus, multimodality imaging appears to play a more significant role in the management of VHD than simply providing a conclusive and comprehensive diagnosis where one imaging technique was inconclusive.

As the management of VHD increasingly expands to include transcatheter interventions for the aortic, mitral, pulmonic, and tricuspid valves, CT imaging has become more critical than ever. Its role in understanding complex anatomical variations and pathophysiology is essential for precise procedural planning and successful outcomes [10]. CT plays a vital role in evaluating valve characteristics to determine whether a patient is suitable for a fully percutaneous intervention or if certain features indicate the need for surgical or hybrid treatment instead.

For example, a CT-based assessment of the risk of left ventricular outflow tract obstruction is essential before determining a patient’s suitability for transcatheter mitral valve replacement. For the aortic valve, the role of CT extends beyond assessing valve anatomy to include the evaluation of potential access routes beyond the traditional transfemoral approach.

Although current evidence remains limited, CMR shows considerable promise in the evaluation of multivalvular disease. CMR provides accurate grading of regurgitant lesions and overcomes many of the well-known limitations of echocardiographic assessment. The use of phase-contrast CMR to quantify flow in the aorta or pulmonary artery is the recommended method for determining regurgitant volume and fraction. In contrast, calculating regurgitant volume as the difference between left and right ventricular stroke volumes obtained from cine sequences can be misleading and may yield inaccurate results, particularly when multiple valvular lesions coexist [11].

CMR is widely regarded as the most accurate modality for quantifying ventricular volumes, wall thickness, and ejection fraction, providing crucial insights into the degree of volume and pressure overload in patients with multivalvular disease, information that can influence the timing of interventions. Beyond structural assessment, CMR enables myocardial tissue characterization, allowing identification of both replacement fibrosis and diffuse fibrosis through late gadolinium enhancement (LGE) and extracellular volume (ECV) mapping, respectively. Emerging evidence suggests that ECV may detect myocardial remodeling earlier than LGE, potentially helping to optimize the timing of invasive treatment [12,13].

CMR also permits evaluation of the aortic valve annulus, both anatomically and functionally, although these techniques remain largely research-focused. Steady-state free precession (SSFP) sequences provide high contrast between blood and myocardium and a favorable signal-to-noise ratio, facilitating accurate measurement of the anatomical annulus. Functional assessment of the aortic valve area can be performed using phase-contrast velocity mapping, which calculates the velocity-time integral across the left ventricular outflow tract and the aortic valve. However, the correlation of these CMR-derived measurements with other conventional diagnostic approaches is still limited [14,15].

Although evidence supporting the prognostic value of multimodality imaging in asymptomatic patients with severe valvular heart disease (VHD) is expanding, most existing studies have focused on symptomatic individuals. Currently, the clinical approach to VHD is evolving toward earlier detection of disease and identification of patients who may benefit from timely intervention. Multimodality cardiac imaging is expected to play a central role in guiding the development of novel therapies aimed at preventing disease progression and preserving left ventricular function.

## 3. Clinical Trials and Proof of Concept Studies

Significant progress in imaging and transcatheter technologies has captured the interest of clinicians, researchers, engineers, device developers, and investors, all working together to develop treatment approaches that are grounded in a deeper understanding of the disease and supported by a team-based approach to VHD care. At the same time, advances in computational tools, new medications, and bioengineering are changing how we diagnose and treat patients with VHD, paving the way for more precise and effective care.

Among the future innovations, new devices which provide a more personalized approach to the anatomy of the patient are expected to provide a solution to patients with complex anatomy or those with certain limitations, such as extensive mitral annular calcification, which still poses a challenge to many surgeons and structural interventionalists [16]. Furthermore, new studies will shed light to the durability of the valves lasting over 20 years [17], and multimodality imaging has highlighted early thrombosis of bioprosthetic valves, making lasting durability a necessity. Another key development in structural heart interventions is the growing use of devices in patients who are at low or intermediate surgical risk, particularly with mitral transcatheter procedures [18]. While transcatheter mitral valve replacement has been primarily employed in high-risk populations, its prime is near approaching, and more trials now will include low- and intermediate-risk patients.

Another step forward is also moving the focus from the valve itself to the myocardium, to avoid myocardial fibrosis and eventually impairment [19,20]. Delaying intervention on an advanced stage of the valvular disease can result in irreversible myocardial injury, adversely affecting long-term outcomes and quality of life, even after the valve abnormality has been treated. By intervening before significant myocardial remodeling and fibrosis occur, clinicians aim to preserve ventricular function, prevent heart failure, and improve survival rates. Hence, we tend nowadays to decide on proceeding with structural intervention, following a heart team meeting, earlier rather than later.

## 4. Multiomics and Metabolomics in SHD

The integration of biomarkers has significantly improved early diagnosis and risk stratification of patients with VHD. Biomarkers indicating myocardial remodeling, inflammation, and calcification processes provide valuable insights into disease progression [21,22].

Simultaneous measurement of hundreds of metabolites in biological fluids or tissue samples with the use of metabolomics technologies enables the characterization of each patient’s unique metabolic fingerprint [23]. This strategy enhances our understanding of the complex metabolic pathways underlying valvular heart diseases, facilitating the identification of novel diagnostic and prognostic biomarkers. Consequently, it enables earlier diagnosis, more accurate risk stratification, and the development of optimized treatment strategies for affected patients.

Moreover, multiomics technologies (including genomics, transcriptomics, proteomics, and metabolomics) uncover new molecular pathways of valve degeneration and dysfunction. Hence, new molecular targets can now be discovered for treatment, designing pharmacological therapies that will prevent or even reverse disease progression [24,25,26].

These great biology advances may significantly transform the therapeutic management of VHD. Early diagnosis of high-risk patients leading to tailored therapeutic approaches, definition of the optimal intervention time, and monitoring of treatment response are all results of this progress.

## 5. Disparities in SHD

The combination of SHD or VHD advances and the awareness of gender and ethnic–socioeconomic disparities has brought to the surface multiple studies examining disparities in presentation, symptoms, or even management. Calcific aortic stenosis and degenerative mitral regurgitation are increasingly recognized as prevalent conditions among elderly women, particularly those with multiple comorbidities. Historically, women with VHD have been underrepresented in many pivotal clinical trials that inform guideline recommendations [27]. This underrepresentation has contributed to delayed surgical referrals in women, often resulting in poorer postoperative outcomes compared with men. This is only one example of the disparities—it is important to focus also on socioeconomic status as well as ethnic disparities, in order to achieve equity in care [28]. Within this spectrum, becoming global is very important, and studies/trials as well as clinical practice should approach everybody, including patients from low-income countries who still suffer from conditions such as rheumatic heart disease. Organizations such as the World Heart Federation are approaching these countries and establishing more effective ways of screening VHD in remote areas where previously large populations would not even obtain an electrocardiogram or echocardiogram [29].

Addressing these disparities requires not only awareness but also demands real action. Research projects integrating all patient groups, advanced diagnostic methods equally available to all patients, and treatment strategies that consider the unique needs of underrepresented groups can achieve true equity in VHD and SHD. Care strategies that make sure no patient is left behind ensure that innovation, expertise, and treatment extend to all corners of the world.

## 6. Frailty in SHD

Nowadays, the assessment of frailty into routine clinical practice is of paramount importance due to the increase in life expectancy. Although traditional risk scores are broadly used, there is a growing need for dynamic tools that include frailty, cognitive function, nutritional status, and social support.

A very important part of the VHD spectrum is the biological versus physiological age, which in a way is translated as frailty. The input of care of the elderly physicians in the heart team is crucial as well as the implementation of various frailty scores [30]. It is important to understand that biological age, which is the age determined by cellular and molecular processes, is substantially different to physiological age, which reflects the body’s functional capacity and the general health status, including cumulative impact of comorbidities and disability. Notably, there are a great number of factors contributing to this gap [31]. Frailty, therefore, becomes a key determinant in selecting the right candidates for advanced SHD treatments, especially transcatheter approaches that often target older and high-risk populations.

Ongoing research studies will further shed light on the appropriate way to address patients with multiple comorbidities who usually go down a pathway of SHD. Development of more accurate frailty scores contributing to improved patient risk stratification and decision-making processes ensure that treatment strategies are incorporating both anatomical and procedural factors and the patient’s overall functional reserve. Future studies should focus on approaches that modify frailty. The improvement of rehabilitation programs before and after procedures along with the nutritional strategies help clinicians to address frailty early, making transcatheter therapies available to patients who might otherwise be considered to be high-risk.

## 7. Congenital Heart Disease and VHD Interventions

The improved survival of patients following surgical repair of congenital abnormalities has resulted in many requiring subsequent valvular interventions over the course of their lifetime. As this growing population of adult congenital heart disease (ACHD) patients ages, the need for multiple interventions, including re-interventions on repaired or palliated lesions, has become an expected part of their lifelong care pathway.

Percutaneous alternatives for treatment and palliation of congenital cardiac lesions traditionally within the realm of surgery have taken prominence over the last decade [32]. Innovations such as transcatheter pulmonary valve implantation (TPVI), percutaneous closure devices for atrial and ventricular septal defects, and hybrid procedures for complex lesions have expanded the therapeutic armamentarium available to both pediatric and adult patients. These advances have reduced the morbidity rates of congenital surgeries due to repeated surgical sternotomies and allowed for staged interventional treatment that combines catheter-based and surgical approaches that respect each patient’s anatomy and physiology. Also, recent developments in device design, such as next-generation transcatheter valves that can accommodate complex right ventricular outflow tract (RVOT) anatomies, biodegradable stents, and patient-specific 3D-printed models for pre-procedural planning, are further pushing the boundaries of what is possible [31,32,33]. Future generations of congenital heart patients should benefit from safer, less invasive, and more durable interventions across their lifespan, and ongoing clinical trials and registries should focus on that approach.

The extension of survival, the minimization of hospital stays, the improvement of re-intervention intervals, and the preservation of ventricular function need close collaboration among ACHD cardiologists, interventionalists, congenital cardiac surgeons, imaging specialists, and nurses.

## 8. Artificial Intelligence

Artificial intelligence (AI) is transforming the landscape of cardiovascular disease diagnosis and treatment by enabling rapid, precise disease identification and personalized care. Machine-learning algorithms can perform enhanced risk stratification and prognostication by using clinical data from electronic health records, complemented by physiological and behavioral data obtained from mobile devices and wearable technologies, as well as patient-generated information from social networks. The automated quantification of valve function and imaging detection of subtle valvular changes has contributed to the early and more precise assessment of VHD. Moreover, AI offers the integration of clinical and imaging biomarkers through simplified algorithms.

In global health settings, where remote access to VHD diagnosis is crucial, AI tools can efficiently analyze large volumes of electrocardiographic and echocardiographic data, facilitating early detection. Early diagnosis of asymptomatic VHD patients can be achieved by AI-enhanced electrocardiogram (ECG) screening and contribute to improved risk assessment and definition of timely intervention [34]. For example, AI-enhanced ECG algorithms can recognize patients with subtle signs of aortic stenosis [35] or mitral regurgitation who would otherwise be undetected until the late-stage of disease where symptoms definitely appear. Moreover, AI-driven remote imaging analysis along with telemedicine can offer expert diagnostic approaches to patients that have never had regular access to echocardiography or a specialist opinion.

Furthermore, AI is increasingly integrated into the treatment pathway. Predictive models can suggest the most suitable transcatheter device based on complex anatomical features, or even predict procedural complications such as leaflet thrombosis, valve malposition, or conduction disturbances, supporting heart team decisions in this way. AI can also contribute to the design of shorter, safer, and more efficient clinical trials that focus on the development of new therapeutic strategies that improve patient outcomes worldwide [36].

While AI holds great promise in the management of patients with VHD, it is also associated with important risks. Algorithmic bias and an over-reliance on automated outputs in place of clinical judgement may lead to inappropriate or even harmful treatment decisions. These risks can be mitigated by ensuring the validation of AI tools across various clinical settings and are applied as supportive tools rather than replacements for physicians’ clinical practice. Continuous clinical monitoring of patients and adherence to ethical standards are essential for a safe and responsible integration of AI into clinical practice [37].

## 9. Multidisciplinary Team Decision-Making in VHD Management

The optimal and effective management of VHD patients relies on the close collaboration among a multidisciplinary team of specialists. Cardiologists, cardiac surgeons, imaging experts, anesthesiologists, geriatricians, interventionalists, nurses, and rehabilitation specialists are valuable members of this team. In addition, ACHD cardiologists, imager specialists, interventionalists, and congenital cardiac surgeons should also be incorporated for the optimal management of ACHD patients [38,39]. Each member offers a comprehensive evaluation of the patient’s disease, offering a unique expertise opinion.

Heart valve teams are essential for providing a high-quality and holistic evaluation of patients with VHD, contributing to complex clinical decisions. Symptoms and comorbidities should be evaluated, as well as laboratory test results including biomarkers, invasive angiography and hemodynamic measurements, and multimodality imaging. Moreover, imaging modalities including CT, CMR, and positron emission tomography are now considered essential components of a comprehensive heart valve center, ensuring accurate diagnosis and informed treatment planning [40]. The heart team should take into account each patient’s overall health status, including frailty and comorbidities. Patient preferences should be respected, and team decisions should consider the functional status, quality of life, and the long-term outcomes, rather than only the anatomical characteristics of the valves [41].

Heart team meetings ought to be held on a regular basis where open communication and shared decision-making will be supported. Moreover, scientific discussion among different specialties encourages ongoing learning with knowledge sharing and improves clinical outcomes. Also, it makes patients feel confident and cared for with treatment truly tailored to their needs.

This approach helps teams stay up to date with advanced diagnostic tools and new treatments guidelines and allows heart valve centers to offer innovative treatment strategies that will respect all patient needs with VHD, leading to more effective future results.

## 10. Going Back the Basics

Above all, going back to the core values of anatomy and physiology in VHD is an important step to understand better the individualized and often complex anatomy encountered in SHD patients. Recent advances, such as the updated classification of tricuspid regurgitation [42], reflecting a more detailed understanding of the valve anatomy, and in addition, the acknowledgement that the underlying etiologic mechanism can categorize both mitral and tricuspid valve lesions (atrial versus ventricular mechanism) [43], provide important insights into the disease pathophysiology and guide improved treatment strategies.

**Figure 1:**
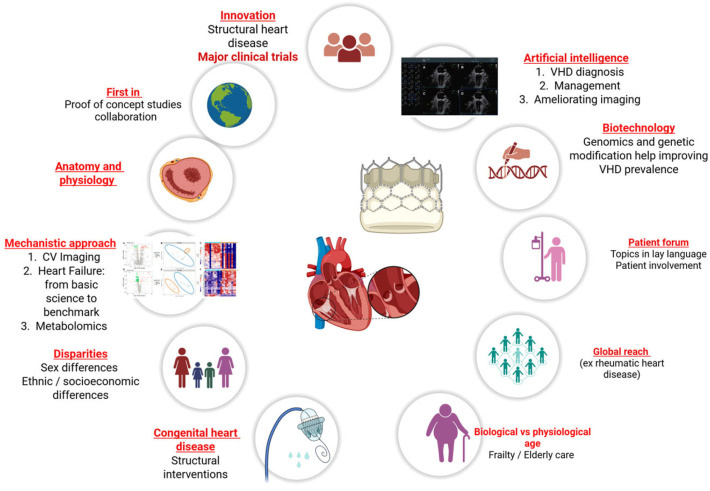
Multiparametric approach of valvular and structural heart disease based on multimodality imaging, mechanistic approaches, patient feedback, frailty, innovative first-in studies, and randomized controlled trials.

In addition, the hemodynamic assessment of valvular lesions [44,45] offers valuable information on the functional consequences of valvular dysfunction and overall cardiac performance. Clinicians who are able to evaluate the myocardial response to the hemodynamic burden of valve dysfunction can easily determine the prognosis and define optimal intervention time for each patient. Multimodality imaging advances with the invaluable incorporation of computational flow technologies provide further details of valve anatomy, flow dynamics, and myocardial mechanics. These developments enable more precise patient selection for the most optimal choice of medical and procedural treatment strategy, improving both short- and long-term outcomes for patients with complex SHD. Valvular and structural heart disease is multiparametric, and while imaging is the first-line tool for diagnosis, we should not forget the multiple aspects that are important for future guidelines and are demonstrated in Figure 1.

## 11. Conclusions

As a conclusion, the field of VHD and SHD is broad, and the future is bright. Many unresolved questions and important needs remain for the care of our future patients. However, continued novel innovations in imaging and valve device technologies along with multidisciplinary collaboration will be the main factors in facing these challenges. A patient-centered approach using the latest scientific advances will shape the next era of VHD management, and this commitment to progress will ensure that patients receive safer, more effective, and longer-lasting treatments tailored to their unique needs.

## Data Availability

Not applicable.

## Abbreviations

VHD	Valvular heart disease
SHD	Structural heart disease
CMR	Cardiac magnetic resonance
CT	Computed tomography
ACHD	Adult congenital heart disease
AI	Artificial intelligence
ECG	Electrocardiogram
2D	Two-dimensional
3D	Three-dimensional
AVA	Aortic valve area
LVOT	Left ventricular outflow tract
DHM	Digital heart model
